# Breadfruit (*Artocarpus altilis*): Processing, nutritional quality, and food applications

**DOI:** 10.3389/fnut.2023.1156155

**Published:** 2023-03-16

**Authors:** Kervyn Ajay Mehta, Yu Chin Rina Quek, Christiani Jeyakumar Henry

**Affiliations:** ^1^Clinical Nutrition Research Centre (CNRC), Singapore Institute of Food and Biotechnology Innovation (SIFBI), Agency for Science, Technology and Research (A*STAR), Singapore, Singapore; ^2^Department of Biochemistry, Yong Loo Lin School of Medicine, National University of Singapore, Singapore

**Keywords:** breadfruit (*Artocarpus altilis*), post-processing, nutritional, food applications, functional properties, ingredient replacer

## Abstract

Breadfruit is an underutilized but highly nutritive crop containing complex carbohydrates while being low in fat. It is also a good source of essential amino acids (leucine, isoleucine, and valine). With a better understanding of breadfruit’s morphology, its potential as a global solution to food security has been gaining popularity. Breadfruit has been forecasted to have a larger amount of suitable cultivable land area compared to major crops such as rice and wheat, making its cultivation more desirable. Due to its highly perishable nature, good post-harvesting and post-processing practices are essential to extend the shelf life of breadfruit for global transportation and consumption. This paper aims to provide a comprehensive review on various processing methods of flour and starch, nutritional significance and new food applications of this novel food staple. In this review, the effects of the different processing and post-processing methods of breadfruit flour and starch have been described, and the nutritional composition and application of breadfruit flour as an ingredient replacer in various food applications have been discussed. It is vital to understand the processing and post-processing methods of breadfruit flour to enhance its shelf-life, physicochemical and functional properties. Furthermore, a compilation of novel food applications has been done to promote its use in the food industry. In conclusion, breadfruit flour and starch are highly versatile for use in numerous food products with added health benefits.

## 1. Introduction

Breadfruit (*Artocarpus Altilis*) belongs to the family of Moraceae and has over 120 cultivars (1). Breadfruit is rich in nutrients and complex carbohydrates while being low in fat and cholesterol (2). It also contains a wide range of amino acids and is particularly high in leucine, isoleucine, phenylalanine, and valine making it a good source of essential amino acids, especially in countries battling malnutrition (3). Breadfruit originated from the Western Pacific, primarily in New Guinea and its neighboring islands (4). It is currently an underutilized, high-yielding crop that grows well in the tropics and sub-tropics such as South America, Caribbean, and Oceania (5).

Presently, what is known as breadfruit consist of *Artocarpus Altilis* and the less common *Artocarpus Altilis* × *Artocarpus Mariannensis* hybrids which were produced from interspecific hybridization, originating in Micronesia (6). The original *A. altilis* comes in various forms, either producing no seeds, two or a few seeds. Whilst the initial generation hybrids take after the *A. mariannensis* parent, the later generation hybrids take after the *A. altilis* and are seedless (7).

Breadfruit’s taxonomy has been a challenge to classify, resulting in erratic binomial nomenclatures (7). This can be attributed to a lack of standardized methodology for data collection, coupled with morphological similarities between breadfruit hybrids and its close relatives, such as Breadnut (*Artocarpus Camansi*) and *Artocarpus Mariannensis* (8). Until recently, a standardized set of morphological characteristics has been developed to distinguish breadfruit and its cultivars from its relatives (7). This systematic method of morphological characterization has allowed for the creation of a genebank to determine cultivars with superior nutritional and sensory qualities for propagation and use in various food applications (7).

With a better understanding of breadfruit’s morphology, its potential as a global solution to food security has been gaining popularity. Breadfruit has been named as a primary crop for further research by the International Treaty on Plant Genetic Resources due to its potential to be a valuable crop for future generations (9). These claims have been supported by Mausio et al. (10)’s model based on future projected climate scenarios. The model predicts that breadfruit will have a larger amount of suitable cultivable land area compared to major crops such as rice and wheat which has been forecasted to decrease in the years to come (10). Furthermore, with its gluten-free properties, the use of breadfruit in developing food products to cater to those suffering from gluten allergies or celiac disease is vastly increasing (11). Despite breadfruit’s fairly long history since the 1950s, breakthroughs were only seen in 2011 and 2015 when the Food and Agriculture Organization (FAO) publicized proposed guidelines on optimal and reliable methods for commercial propagation (3, 12). These guidelines stimulated an increase in the awareness of the potential food product application of breadfruit.

A wealth of studies focusing on individual topics such as breadfruits’ functional, nutritional, and chemical properties, and food application of native and processed breadfruit flour and starches have been documented. In contrast, this paper aims to provide a comprehensive review on various processing methods (starches and flours), nutritional significance, health benefits, and new food applications of this novel food staple.

## 2. Post-harvesting practices of breadfruit

Breadfruit is a highly perishable fruit that requires good post-harvesting practices to extend its shelf life for global transportation and consumption. Its high respiration rate and susceptibility to pests such as fruit flies and mealybugs make it crucial to ensure that proper post-harvesting methods are utilized. Several methods have been explored in optimizing post-harvesting of breadfruit. Heat treatment using water and air as a disinfection step against pests for breadfruit has shown promising results, without negatively impacting the quality of breadfruit (13). There are other forms of traditional post-harvest processing recommendations with regards to maturity and quality indices, temperature and controlled atmospheric conditions, and potential pathological and physical disorders. These authors found that storage of harvested breadfruit 13 ± 1^°^C with a relative humidity of 85–95% and minimal exposure to ethylene prolongs the shelf-life of breadfruit (14). The novel usage of semipermeable coating was explored by Worrell et al. (15) who reported reduced fruit softening, albeit off - odors and greater discoloration of the flesh of breadfruit. Another study showed that controlled atmospheric storage in 5% CO_2_ and 5% O_2_ at 16^°^C of breadfruit reduced skin discoloration significantly and extended shelf-life of breadfruit to 25 days compared to 8 days of untreated breadfruit (16). These studies that have been done are vital steps that the food industries have to first understand in order to optimize the quality of breadfruit, determine its optimal storage condition and ultimately extend its shelf life.

## 3. Breadfruit pulp processing

**FIGURE 1:**
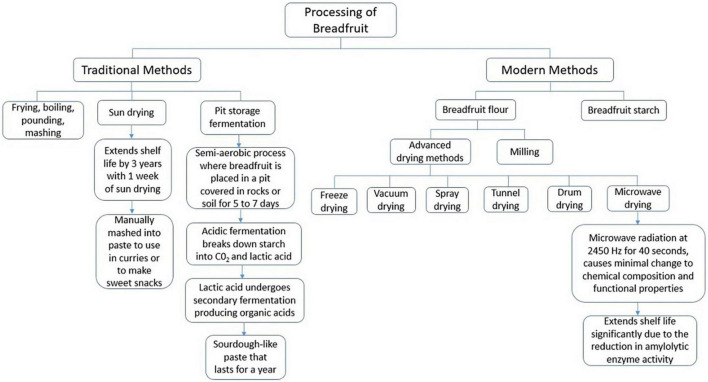
Traditional and modern methods for processing breadfruit pulp.

In view of the increasing interest in breadfruit as a major staple, a key consideration is its processing and its food application potentials. Breadfruit is a versatile food as its fruit, seeds, leaves and flowers are edible. However, research regarding the processing and food application potentials of its seeds, leaves and flowers are still limited, hindering its use for commercial purposes. Presently, only the pulp of breadfruit is eaten accompanied with a sauce or may be used as a carbohydrate staple. The skin is undesirable to taste resulting in its valorization efforts to be used as an absorbent for toxic dyes (17). A flow diagram summarizing the traditional and modern methods of processing breadfruit is illustrated in Figure 1.

### 3.1. Traditional methods of processing

Breadfruit is processed differently in various regions. The consumption of breadfruit pulp was a staple in the diet of people mainly living in Oceania while in regions such as South America and the Caribbean, its consumption was stigmatized and associated with poverty and slavery (2). In South America and the Caribbean, the main processing methods of breadfruit were limited to frying, boiling, pounding, and mashing to make dishes such as fritters and porridges. These cooking methods were adopted to improve the sensory properties of breadfruit. It was later discovered that these cooking methods also reduced the anti-nutritional compounds present in breadfruit (18). Stachyose, hemagglutinin and raffinose are compounds which could interfere with the digestive process during consumption (18).

Several methods mainly developed by Pacifists living in Oceania, have been used to prolong the shelf life of breadfruit. One method involving traditional drying under the sun for a week after being diced and cooked, extends the shelf life of breadfruit for up to 3 years (19). This can be manually mashed into a paste to make a sweet snack or cooked in curries (20). This drying process is necessary to halt the fermentation process and the growth of bacteria and fungi (21). Another method of extending the shelf life of breadfruit involves pit storage fermentation. This method uses a semi-anaerobic process in which fermentation of peeled and cored breadfruit occurs when it is placed in a pit covered in rocks or soil for 5–7 days (22). The acidic fermentation process causes pH within the breadfruit pulp to drop below four, breaking down starch into carbon dioxide and lactic acid *via* a complex process (4). The lactic acid produced undergoes secondary aerobic fermentation leading to the production of organic acids like butyric and acetic acids (23). The end result of pit storage fermentation is a sour dough-like paste which can last for a year (23). This fermented paste is traditionally washed to reduce the acidic taste before pounding and cooking for use in dishes.

### 3.2. Modern methods of processing

**FIGURE 2:**
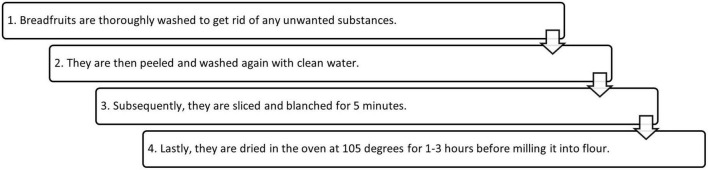
Process of manufacturing breadfruit flour.

Currently, breadfruit pulp is used in either the breadfruit flour and/or as an isolated starch variant. They are more stable, and have a greater versatility as an ingredient in formulated food products. A simplified process of breadfruit flour is shown in Figure 2.

#### 3.2.1. Processing of breadfruit flour

In recent times, breadfruit flour has been gaining interest for its multi-faceted use in various vegan and gluten-free industrial food applications (24). Its taste preference amongst consumers has been rising (25). Breadfruit flour is typically made from cooked, dried breadfruit to reduce anti-nutritional compounds before milling and sieving (18). There is limited research looking at optimal processing methods to reduce the anti-nutritional contents present in breadfruit. Besides cooking and boiling, the usage of microwave radiation has been explored by Arinola and Akingbala (26). The authors found that microwave radiation caused minimal changes to chemical composition and functional properties of breadfruit while extending the shelf life of breadfruit flour significantly due to a reduction of the activity of amylolytic enzymes present (26).

Over the years, with advancement in technology, the process of drying breadfruit flour has shortened tremendously–from a week of sun drying to just a few hours of drying in oven and heating chambers at temperatures 40 to 60^°^C (19). While retaining a similar shelf-life duration, heating chambers and oven drying has been reported to show significant improvements in proximate reduction in anti-nutritional factors, nutritional, antioxidant, and functional properties of breadfruit compared to sun drying (19).

Heating temperature and drying duration has been determined as crucial parameters in the drying process, significantly influencing the functional and chemical properties of breadfruit flour. A better retention of nutritional qualities of breadfruit flour (using matured fruit) was noticed when the oven drying method was used (19). Sari and Lestari found that the optimum breadfruit flour production in terms of appearance was achieved with a temperature of 60^°^C and a drying time of 100 min (21). At 60^°^C, minimal browning of the flour *via* Maillard reaction occurs and as temperature rises, more Maillard reaction was seen. A greater rate of browning by Maillard reaction could indicate a decrease in flour quality (21). Furthermore, when temperatures above 60^°^C was utilized, the texture of the flour became undesirable and coarser which could limit the functionality for use in food applications (21). This could possibly be attributed to breadfruit flour having a low gelatinization temperature between 70 and 80^°^C (27). Usage of higher heating temperatures have reported greater starch retrogradation of breadfruit flours (27). With similar drying conditions (60^°^C, 100 min), Tijani et al. (28) found the functional and pasting properties such as solubility, bulk density, swelling power, water absorption capacity, dispersibility, and pasting characteristics to be the most desirable for use in various food applications. At constant heating temperatures, increasing in drying time did show greater flour solubility but lower dispersibility, gelation capacity, and yield (21, 28, 29). Optimal drying conditions of breadfruit was also modeled in a study, whereby a combination of heating, drying and velocity chambers was utilized, to chart the ideal drying time based on air velocity and temperature parameters (30).

More recently, advanced drying methods of breadfruit such as freeze drying, vacuum drying, spray drying, tunnel drying, and drum drying have been sparsely explored (31). Amongst all the other drying methods, microwave drying has shown to be more homogenous, rapid, energy efficient, and ultimately is able to produce a product of improved physical quality (32). Microwave-vacuum drying i.e., the combination of microwave and vacuum drying further enhances the color, texture, and flavor of many dehydrated fruits (33). Taruna et al. (34) found microwave power as the more crucial factor in significantly influencing the physical quality of breadfruit flour compared to the duration of grinding. Tunnel drying and freeze drying resulted in greater solubility of breadfruit flour whilst ensuring similar textural properties compared to breadfruit that is milled, cooked, steamed, and oven dried (35). Studies have shown that low temperature-controlled vacuum drying produce breadfruit flour of a low water content (approximately 2%) with good reconstitution capabilities (36). Additionally, low temperature-controlled vacuum drying requires less drying time and operational cost compared to freeze drying breadfruit (36). Spray drying, on the other hand, also has a lower operational cost, shorter processing time and a higher yield compared to freeze drying (37). The limitation, however, is the increased chance of heat injury. The inlet temperature of a spray dryer ranges from 200 to 500^°^C which can negatively alter the chemical and functional properties of breadfruit flour such as its solubility, emulsification, and fat retention (27). The combination of drying techniques such as microwave-vacuum drying i.e., the combination of microwave and vacuum drying have shown to further enhance the color, texture, and flavor of many fruit flours and has yet to be explored with breadfruit flour (33). Additionally, novel drying techniques such as refractance window dehydration, superheated steam drying, high electric field drying, infrared drying, and heat drying pump which have shown promise for drying of various food items in the industry (38). Such methods should also be explored with breadfruit flour to improve its functional and physicochemical properties.

Milling of breadfruit flour and its impact on particle size, functionality, and physiochemical properties have not been explored in the literature. This is a potential area for further research as various milling methods such as jet, disk and ball milling have shown to significantly influence these factors in various grains like barley and rye (39). Native breadfruit flour unfortunately has shown to be limited in the groups of industrial food applications that it can be utilized in Sunarti et al. (31). It has low swellability and solubility at room temperature. Thus, post-processing methods are essential and commonly used to improve the functionality and physicochemical properties of breadfruit flour and tailor it to respective food application uses.

#### 3.2.2. Processing of breadfruit starch

Starch isolation from amylaceous breadfruit as a food additive to control the stability, uniformity and texture of various food applications and as a sweetener is becoming popular (40). The amylopectin and amylose content of breadfruit starch is approximately 22.52 and 77.48%, respectively (41). Breadfruit starch has been observed to have superior functionality over wheat, rice and cassava flour in terms of viscosity, oil, and water binding capacity, and swelling power (3, 27, 42). Breadfruit starch is small, irregularly shaped (polyhedral, spherical, and elliptical) and ranges in size from 3.0 to 7.9 μm (43). Breadfruit starch is typically extracted through a multi-step process described in detail by Loos et al. (44). In short, breadfruit flour is first made into a slurry by blending the flour with water. The precipitate is then obtained from the centrifuged slurry and finally, the precipitate is dried (44). This method produces a moderately high starch yield of approximately 14–18% (45). Ultrasound extraction of breadfruit has been attempted as well. When optimized, it can potentially be used in a wide range of colloidal systems for extending shelf life and as fat mimetics *via* a physical procedure without any chemical intervention (42). However, utilizing breadfruit starch in its native form without any post-processing has limited practical potential due to their inferior water solubility, instability when processed under high shear and/or high temperature resulting in a greater tendency of retrogradation and syneresis (46). This can be explained by the high amounts of amylose present which binds easily to lipid molecules, thereby forming amylose-lipid complexes. These amylose-lipid complexes enhances the absorption of oil and can affect the quality of food products (47). Thus, like breadfruit flour, post-processing methods are also commonly utilized for breadfruit starch.

Overall, the modern processing method is an improved version of the traditional method. It requires a shorter duration and produces a product with better quality. With processing, breadfruit flour and starch have a longer shelf life compared to its native form thus, allowing for use in various food applications. However, post processing of breadfruit flour and starch is needed to improve functionality to produce food products with better acceptability.

### 3.3. Post-processing of breadfruit flour

**FIGURE 3:**
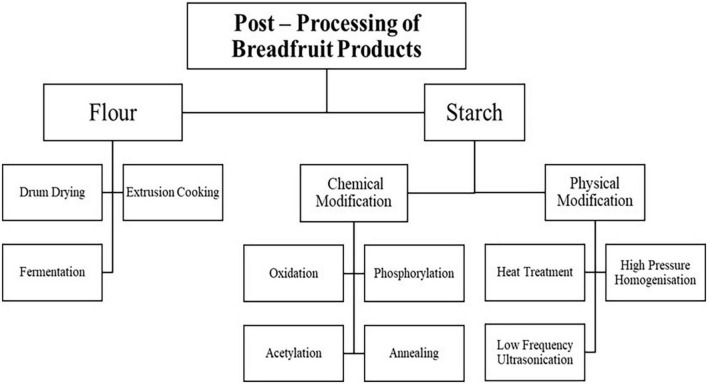
Post-processing of breadfruit products.

Post-processing of breadfruit flour is common in industrial settings to improve shelf life, functional and physicochemical properties. Minimal technical advancement has been made to post-harvest handling of breadfruit to increase its shelf life and this poses a challenge to its usage in various food applications. Hence, there is a need for further exploration of post-processing technology for breadfruit. In this review, some of the common post-processing methods of breadfruit flour including drum drying, extrusion cooking and fermentation will be discussed in the subsections below. An overview of the post processing methods of breadfruit flour and starch is shown in Figure 3.

#### 3.3.1. Drum drying

Drum drying is a form of steam drying. During the process of drying, breadfruit flour pregelatinized the starch component of breadfruit flour which significantly affects the functional and physicochemical properties of the flour (48). Due to the disruption of starch granules, pregelatinized flour is able to be better absorbed and showcased greater viscosity in water, regardless of its temperature (48). Drum drying is rapid due to its efficient heat and mass transfer. Furthermore, it does not require a large amount of area to operate and allows for temperature control when enclosed in a vacuum chamber (49). Drum dried breadfruit flour displayed superior rehydration properties (improved water solubility) indicating good porosity due to the removal of water through boiling. Additionally, drum dried breadfruit flour exhibited lower gelatinization temperature, and better swelling power in solutions regardless of temperature without influencing the chemical composition of the flour (31, 49). Numerous variables during drum drying such as rotational speed, steam pressure, film thickness, and feed material characteristics tends to influence functional and physicochemical properties of breadfruit flour (31, 49). An initial research by Sunarti et al. (31) determined the effects of rotational speed and slurry concentration on the characteristics of breadfruit flour and they found that low rotational speed of the drum caused an increased in gelatinization of flour. There is a need for further research to optimize drum drying conditions and alternative steam drying methods to improve the functionality of breadfruit flour. A potential step that could be beneficial to the optimization of breadfruit flour could be using the superheated steam drying method to pregelatinized flour (50). Superheated steam drying has shown to be advantageous for nutrient preservation due to the lack of oxygen steam in superheated which prevents the decomposition of easily oxidized nutrients. Furthermore, it has shown to be faster and more cost effective than most drying methods (51).

#### 3.3.2. Extrusion cooking

Extrusion cooking of breadfruit flour is another method of pregelatinized flour using both mechanical and thermal energy. It is a quick and low-moisture process without the use of additives and chemicals (52). Extrusion cooking differs from drum drying as it is more severe in the extent of depolymerization of amylose and amylopectin *via* random chain splitting resulting in greater decrease of average molecular weights (52). Furthermore, extrusion cooking of breadfruit flour ensures amylopectin/amylose ratio is similar to its native flour whereas drum drying results in the flour being enriched with amylose (53). Thus, the different processing methods will result in varying functional properties of breadfruit flour. The starch gelatinization and protein denaturation caused by extrusion cooking has shown to influence functional properties such as thermal, hydration, pasting, and rheological properties (54). These factors are affected by various extrusion parameters and its intensity which include moisture content, feed rate, temperature of barrel, and screw speed (55). Extrusion cooking of breadfruit flour has shown to retain its starch and protein content however, a significant decrease in lipid and a significant increase in ash content was observed (56). Extruded breadfruit flour using low specific mechanical energy highlighted greater water-binding capacity and lower solubility compared to flour extruded using high specific mechanical energy. This could possibly be attributed to the starch molecules undergoing lower shear fragmentation (57). Currently, most research relating to extrusion cooking of breadfruit flour revolved around hot extrusion using a twin screw extruder and its potential as gelling agent in protein/starch food systems or as breakfast cereal or snack (56). Further research needs to be done to obtain an optimal method using various extrusion parameters on the functional and physicochemical properties of breadfruit flour (52). It has also been reported that mild extrusion conditions i.e., high moisture, low temperature, and low residence time ameliorates nutritional quality of foods (58). Hence, future research can focus specifically on investigating the retention of nutritional content of extruded breadfruit flour.

#### 3.3.3. Fermentation

Fermented foods has been fast gaining traction amongst consumers due to their proposed health benefits (59). The use of various lactic acid bacteria such as *Lactobacillus plantarum, Lactobacillus acidophilus*, and *Lactobacillus casei* with breadfruit flour as the substrate has been explored (60). Breadfruit flour was found to be an acceptable substrate for fermentation resulting in good cell viability, thereby playing a prebiotic role. Further research is required to study how fermentation affects the macro- and micronutrient content of breadfruit flour and survivability of probiotic microorganisms in fermented breadfruit flour products.

### 3.4. Post-processing of breadfruit starch

Chemical and physical modification of breadfruit starch are common post-processing methods. Various forms of chemical and physical modifications such as oxidation, phosphorylation, acetylation, annealing, and heat treatment have been explored to improve functionality and physicochemical properties of breadfruit starch (61, 62). All forms of modifications tend to decrease peak viscosity, pasting temperature, hot, and cold paste viscosity of the starch except heat-moisture treatment where pasting temperature of starch increases (61, 62). Heat-based treatments resulted in an increased in pasting temperatures due to the formation of a stronger crystalline structure (complex bonds between amylose in the crystalline region and amylopectin in amorphous region) which is more resistant to swelling and rupture (62). Heat-moisture treatment of starch promotes springiness of the starch (the ability for the starch to bounce back to its original position after a compression), enhanced crumb textural properties and a higher specific volume when incorporated into baked products (63).

#### 3.4.1. Chemical modification of breadfruit starch

Modern chemical methods such as cross-linking has been explored by Amorim et al. (64). They found that using a combination of sodium tripolyphosphate and sodium trimetaphosphate produced starch granules that were more resistant to swelling at higher temperatures with greater solubility and water absorption capacity (64). Modified cross-linked breadfruit starch has shown to be desirable for use in heat sterilized food, up to five freeze-thaw cycles, and has shown to produce type 4 resistant breadfruit starch which can function as a food stabilizer and thickener (65). Anwar et al. (66) produced modified breadfruit starch with octenyl succinic anhydride which showed tremendous emulsifying properties to stabilize emulsions containing polyunsaturated fatty acids.

#### 3.4.2. Physical modification of breadfruit starch

High pressure homogenization and low frequency ultrasonication have been utilized to further improve modified breadfruit starch with octenyl succinic anhydride. This was done to further improve emulsion stability to over 42 days making it a more desirable stabilizer alternative to be used in emulsion-based products such as salad dressings and mayonnaise (67). Anwar et al. (66) reported that high pressure homogenization produced a more uniform mean emulsion droplet size and emulsion stability. This covered a wide range of oil loads compared to low frequency ultrasonication where mean emulsion droplet size varied more. However, it was more efficient when the ratio of the dispersed phase in the emulsion is lesser (67). Furthermore, several methods were observed to be able to produce a greater proportion of type 3 resistant breadfruit starch. These methods include gelatinizing breadfruit starch via boiling at 90^°^C for 3 min, pullulanase debranching at 60^°^C for 24 h, autoclaving, cold storage cycle for 24 h at 4^°^C and an acid hydrolysis for purification (68). Type 3 breadfruit resistant starch has shown superior functionality in terms of high pasting temperature, thermal stability and improved sensory properties after cooking, while providing desirable health benefits. Some of the health benefits encompasses greater satiety, bowel health, lower energy intake and improved insulinemic and glycemic response (68).

## 4. Nutritional composition of breadfruit flour and starch

### 4.1. Nutrient composition and toxicity of breadfruit

Breadfruit has been marketed as the top 25 superfoods in the world that has the potential to play a role in the management and reducing the risks of developing chronic non-communicable diseases such as diabetes, hypertension, and obesity (69). The distinctive traits of a breadfruit is its low energy density (110.18–350.75 kcal/100 g) and yet highly nutritive content. Breadfruit is not only a good source of carbohydrate, dietary fiber, and protein, it also contains substantial amounts of micronutrients such as calcium (19.70–314.47 mg), potassium (361.60–1393.50 mg), iron (0.50–28.13 mg), vitamin C (8.57–16.35 mg), and B3 (1.11–3.34 mg) (5, 70). It is also high in antioxidant as it contains high levels of carotenoids, mainly from beta carotene (228.00–2,751.00 μg) and lutein (253.93–1,011.00 μg). An extensive profile of nutrient composition of the various forms of breadfruit are listed in Tables 1, 2 below. Breadfruit is a better source of protein compared to cassava and has similar protein content to banana and sweet potato (70). There is a lack of studies in terms of the toxicity level of breadfruit flour and starch. Most of the studies focus on the acute toxicity of breadfruit bark and leaves which revealed no toxic reaction or mortality (71, 72). However, Vianney et al. (73) reported that the half maximal effective concentration (EC_50_) toxicity value of breadfruit pulp extract was greater than the effective concentration for the antioxidant activity from the various assays, which confers the food safety of breadfruit pulp. Kumaraswamy and Senthamarai (74) reported that minerals, trace and heavy metal composition of breadfruit pulp using ethanol extraction was within the recommended dietary allowances (RDA) for adults established by USA National Institute of Health (NIH). Furthermore, dichloromethane, hexane, and methanol extract of breadfruit pulp displayed high antioxidant activity and no toxic reaction (71).

**TABLE 1:** Mean reported values for breadfruit (*Artocarpus altilis* and hybrids) proximate analyses per 100 g sample.

Proximate analyses	Fresh (5, 114)	Cooked (5)	Flour (5, 6, 12, 27, 115)	Starch (61, 107, 116, 117)
Ash (%)	2.70 ± 2.69	NA	2.85 ± 2.25	0.41 ± 0.16
Moisture (%)	65.16 ± 24.68	70.73 ± 12.21	7.66 ± 6.30	11.43 ± 3.06
Dry matter (%)	43.37 ± 33.38	31.60 ± 21.50	87.87 ± 9.27	NA
Energy (Kcal)	146.50 ± 81.30	110.18 ± 34.92	350.75 ± 42.55	NA
Total carbohydrates (g)	33.20 ± 21.75	25.71 ± 9.26	77.09 ± 12.50	82.83 ± 2.34
Fats (g)	1.77 ± 2.11	1.25 ± 2.07	2.85 ± 3.37	0.60 ± 0.10
Protein (g)	2.02 ± 1.88	3.04 ± 4.68	5.16 ± 4.72	1.71 ± 1.37
Crude fiber (g)	4.03 ± 2.59	3.49 ± 2.60	10.31 ± 10.40	NA
Insoluble fiber (g)	14.35 ± 15.91	11.20 ± 12.45	34.90 ± 38.75	NA
Soluble fiber (g)	1.25 ± 1.28	3.60 ± 5.09	5.85 ± 7.99	NA

**TABLE 2:** Minimum and maximum carotenoid and vitamin content for **breadfruit** (*Artocarpus altilis* and hybrids) per 100 g sample.

Vitamins and mineral content	Fresh (5, 114)	Cooked (5)	Flour (5, 6, 12, 27, 115)
Total carotenoids (μg)	1,884.50 ± 2,665.09	630.00 ± 890.95	3,274.50 ± 4,630.84
Alpha carotene (μg)	130.00 ± 183.85	71.00 ± 100.41	269.00 ± 380.42
Beta carotene (μg)	861.73 ± 1,698.88	228.00 ± 426.84	2,751.00 ± 3,890.50
Beta cryptoxanthin (μg)	1.65 ± 2.33	6.70 ± 5.83	10.50 ± 14.85
Lutein (μg)	253.93 ± 379.35	279.83 ± 416.92	1,011.00 ± 1,429.77
Lycopene (μg)	24.50 ± 34.65	15.17 ± 13.53	50.00 ± 70.71
Zeaxanthin (μg)	30.00 ± 42.43	35.00 ± 49.50	91.00 ± 128.69
Folic acid (μg)	0.65 ± 0.92	0.61 ± 0.54	1.55 ± 2.19
Vitamin B1 (mg)	0.18 ± 0.09	0.13 ± 0.05	0.42 ± 0.14
Vitamin B2 (mg)	0.07 ± 0.03	0.05 ± 0.02	0.19 ± 0.19
Vitamin B3 (mg)	1.28 ± 0.40	1.11 ± 0.47	3.34 ± 1.47
Vitamin C (mg)	16.35 ± 7.28	11.49 ± 7.74	8.57 ± 12.33
Boron (mg)	0.52 ± 0.00	0.39 ± 0.19	1.27 ± 0.00
Calcium (mg)	28.98 ± 14.53	19.56 ± 8.23	314.47 ± 425.74
Chlorine (mg)	1.00 ± 1.41	0.80 ± 1.13	2.45 ± 3.46
Cobalt (μg)	0.55 ± 0.78	0.45 ± 0.64	1.35 ± 1.91
Copper (mg)	0.14 ± 0.10	0.27 ± 0.21	18.18 ± 27.23
Iron (mg)	10.90 ± 22.98	0.50 ± 0.44	28.13 ± 38.34
Magnesium (mg)	34.35 ± 20.56	25.33 ± 7.79	103.17 ± 95.05
Manganese (mg)	0.14 ± 0.16	0.13 ± 0.12	1.36 ± 1.80
Nickel (mg)	0.04 ± 0.06	0.03 ± 0.04	0.10 ± 0.13
Phosphorus (mg)	50.28 ± 57.86	29.44 ± 9.39	924.75 ± 983.68
Potassium (mg)	759.60 ± 914.75	361.60 ± 104.59	1,393.50 ± 1,255.08
Sodium (mg)	12.06 ± 11.72	24.05 ± 30.69	320.00 ± 364.39
Sulfur (mg)	25.50 ± 7.78	20.00 ± 5.66	62.50 ± 19.09
Zinc (mg)	0.18 ± 0.20	0.09 ± 0.05	4.55 ± 6.75

#### 4.1.1. Carbohydrates and dietary fiber

Breadfruit has a low glycemic index (GI) and has been touted as a traditional, diabetic-friendly fruit (75). The GI is an index used to categorize carbohydrate foods into different groups (low, medium, high) based on their ability to raise blood glucose levels (76). Breadfruit and its flour were determined by various researchers to be lower in GI than wheat and rice flour (77). This could be attributed to its high dietary fiber and amylose content which potentially aids in slowing down glucose absorption in the gastrointestinal tract (78). Cooked breadfruit displayed a lower GI which is more beneficial to combating diabetes (79). During fermentation, dietary fiber in breadfruit produces short chained fatty acids which can lower cholesterol production and in turn decrease triglycerides and low-density lipoprotein cholesterol content (80). The lactic acid bacteria and soluble dietary fiber found in fermented breadfruit makes it symbiotic, containing both pre- and probiotics which are beneficial for gastrointestinal health (80).

#### 4.1.2. Protein

Breadfruit is a source of high quality protein and is made up of a complete protein, specifically rich in valine, isoleucine, phenylalanine and leucine (3). However, certain cultivars of breadfruit have a higher overall protein and essential amino acid content. Jones et al. (12) found that the cultivar, Ma’afala had the highest protein variety and essential amino acid content. Ma’afala displayed a higher percentage of essential amino acid content as a proportion of the whole protein when compared to soybean, indicating superior protein quality (3). Additionally, digestibility of breadfruit protein was found to be higher when compared to wheat protein in *in vitro* studies (76).

#### 4.1.3. Vitamins, minerals, antioxidants, and phytochemicals

Breadfruit contains β-carotene and other carotenoids which confer protection against vitamin A deficiency, cancer, and heart diseases (81). Additionally, prominent amounts of phosphorous, calcium, magnesium, copper, and potassium are also present in the fruit which is aids in increasing bone strength, lowering the risk of cardiovascular diseases, maintaining and developing of the immune system (82). Several researchers reported calcium as the most abundant mineral in breadfruit pulp which can potentially reduce calcium deficiency and improve the growth and development of bones, especially in infants of developing countries (6, 83–85). Studies by Nur Arina and Azrina as well as Soifoini et al. (83) reported high antioxidant activity ranging from 2.2 to 6.4 mmol Fe^2+^/kg_*DW*_ using a ferric reducing antioxidant power (FRAP) assay which is higher than those found in Jackfruit making it a viable functional food (86). Breadfruit has a unique phytochemical profile as it produces over 70 phytochemicals from the pathway, mainly flavones and flavonoids (87). Handa et al. (88) found that many of breadfruit’s phytochemicals confer beneficial biological activity such as anti-bacterial activity, platelet aggregation, anti-fungal properties, anti-tumor agents, and inhibition of leukemia cells. Cinnamic acids and chlorogenic acid were the main phytochemicals found in breadfruit pulp (89, 90). Cinnamic acid undergoes rapid metabolism and have shown low bioavailability in the human body making the actualization of therapeutical effects difficult (91). Chlorogenic acid, on the other hand, plays a pivotal role in lipid and glucose metabolism regulation and related disorders such as obesity and diabetes (92). Breadfruit, particularly the unseeded variety has shown to be a good source of vitamin C which helps in boosting immunity (82). Interestingly, Soifoini et al. (83), reported that monoterpenes, specifically limonene, phellandrene and sabinene were some of the most prominent molecules detected in breadfruit pulp. With studies displaying the pharmacological potential of monoterpenes in the inflammatory disease treatment, more research can be done to further justify and modernize the traditional use of breadfruit as an inflammatory disease remedy (87, 93).

### 4.2. Nutritional differences between breadfruit cultivars

Numerous studies have shown that there are significant variations in nutritional composition between breadfruit cultivars (94). A recent study by Kehinde et al. (46) showed differences in characteristics of various cultivars of breadfruit flour and starch. Interestingly, they found a particular underutilized cultivar (Yuley) for commercial propagation that is rich in starch content, and had the highest content of both soluble and insoluble dietary fiber (46). The Yuley cultivar performed significantly better in nutritional properties than the most popular cultivated commercial cultivar, Ma’afala (46). Protein, carbohydrate and fiber content also varied between breadfruit cultivars ranging between 10–17, 57–75, and 1–3%, respectively (95).

## 5. Food applications of breadfruit flour and starch

Industrial food application of breadfruit is pertinent for the masses to adopt breadfruit as a food source. The nutritional composition of breadfruit, its functional, technological (specifically in its flour and starch form) and physicochemical properties have shown to positively benefit the production of healthier food products using breadfruit for consumers (6, 95). However, despite its superior retrogradation properties to lengthen shelf life, complete substitution of breadfruit flour in certain food matrices may not be optimal due to by its low protein content (29). Listed below are some examples of the use of breadfruit flour and starch in baked goods, confectionaries, carbohydrate staples, beverages, dairy products and meat products. Table 3 shows a summary of key findings for the application of breadfruit.

**TABLE 3:** Summary of key findings on various applications of breadfruit.

Application of breadfruit	Key findings
1. Baked goods	Baked bread substituted with 10–15% of breadfruit flour has acceptable sensory properties i.e., no significant differences in terms of crust, aroma, shape, chewiness, hardness and springiness compared to the normal wheat flour bread. However, it is slightly lower in moisture and volume.
	Biscuit when substituted with 15% breadfruit flour displayed a significant difference in sensory attributes compared to the whole-wheat flour biscuits. However, the biscuits were acceptable with 5% soy protein and 10% breadfruit flour.
	Cookies made with pure breadfruit flour had similar sensory properties to pure wheat flour cookies.
	Pressed cookies and pie crust (without filling) with pure breadfruit flour reported higher sensory acceptance ratings with better crunchiness.
	Cakes substituted with 10–30% showed no significant affect to sensory attributes. However, full substitution of breadfruit flour had undesirable effects on texture.
	Muffins made with 15% breadfruit flour and 5% breadfruit starch displayed similar organoleptic properties.
2. Carbohydrate staples	Pasta made using breadfruit flour, tapioca starch, salt, psyllium powder, xanthan gum and coconut oil showed similar acceptability to the normal wheat pasta.
	Noodles substituted with 30% breadfruit flour showed no significant differences in organoleptic quality. However, noodles made with 20% breadfruit starch and 80% wheat flour showed better proximate, functionality and organoleptic attributes compared to 100% wheat flour noodles. Full substitution of breadfruit flour negatively impacted perception on slipperiness and glossiness.
3. Beverages and dairy products	Probiotic beverages made with 7% breadfruit flour, 1% inoculum, and 15% sugar after fermentation at 30^°^C for 48 h produced the best tasting beverage.
	Breadfruit milk alternatives and probiotic yogurt with soy milk and 4% breadfruit flour with *Bifidobacterium bifidum* (ATCC 11883) and *Lactobacillus acidophilus* showed promising results.
4. Meat analogs	Soy protein gels incorporated with 4% extruded breadfruit flour did not affect processing yields and helped improve color and texture of protein/starch food systems.
	Beef meat emulsions combined with 3% extruded breadfruit flour did not affect redness value of cooked meat emulsions.

### 5.1. Baked goods

Breadfruit flour and starch has been utilized completely (100%) and in blends (substituting a portion of the original ingredient) to produce bread, biscuits and other baked goods. In order for food products to be successfully marketed and sold in the food industry, it is vital that these modified baked products taste good or better than the original product. Several studies have shown that bread when substituted with 10–15% of breadfruit flour displayed acceptable sensory properties (96, 97). When 10% of breadfruit flour was used, no significant difference in terms of crust, aroma and shape was observed, compared to a pure wheat flour bread (98). Zakaria et al. (99) showcased that up to 15% breadfruit starch substitution had no textural differences in terms of chewiness, hardness, and springiness but slightly lower moisture and volume compared to a pure wheat flour bread.

However, the threshold for producing a biscuit with breadfruit flour that is sensorily acceptable is lower. Olaoye et al. (18) observed that a 15% breadfruit flour substitution produced biscuits that were significantly different in sensory attributes compared to whole wheat flour biscuits. This is an important observation and is crucial in the application and formulation of biscuits. Nonetheless, when biscuits had a blend of 5% soy protein and 10% breadfruit flour, they displayed acceptable sensory properties (96). Cookies, on the other hand, were more versatile. Pure breadfruit flour cookie showed similar sensory properties compared to a pure wheat flour cookie despite it containing significantly more resistant starch and amylose (100). In addition, low-fat high-protein cookies made with 20% breadfruit flour was not significantly different in sensory properties compared to a pure wheat flour cookies (101). A total of 10–30% breadfruit flour substitution for use in cakes have shown to not significantly affect sensory attributes (102). Muffins made with 15% breadfruit flour and 5% breadfruit starch have shown similar organoleptic properties compared to 100% wheat flour muffins (103). Complete substitution of wheat flour using breadfruit flour in pressed cookies and pie crust (without filling) showed significantly higher sensory acceptance ratings with better crunchiness. However, a complete substitution of wheat flour in battered cake significantly lowered sensory acceptance ratings due to its impact on the cake texture (104). A shelf-life study conducted by Ranjini et al. (105) demonstrated that as the percentage of breadfruit flour substituting wheat flour increases, the initial moisture content and the rate of moisture absorption also increases. This may then affect stability and microbial growth. However, after 12 weeks, the overall plate count determined *via* microbial analysis was still within the acceptable limit for cookies produced using 100% breadfruit flour with the least amount of changes reported in organoleptic properties compared to other treatments (29). Further physiochemical research should be explored to determine the impact of breadfruit flour in the food matrices of different baked goods. These noteworthy observations can conceivably be the future of healthier and tastier cookies, cakes and muffins.

### 5.2. Carbohydrate staples

Carbohydrate staples such as pasta and noodles are usually made from wheat flour. Some of the Orecchiette type pasta produced using breadfruit flour, tapioca starch, salt, psyllium powder, xanthan gum, and coconut oil through a pasta extruder have showed similar acceptability to that of wheat pasta (11). No significant differences in organoleptic quality was observed when 30% breadfruit flour was incorporated with wheat flour to produce wet noodles with similar expansion, water absorption, tensile strength, greater dietary fiber content (106). In addition, Akanbi et al. (107) reported that noodles manufactured with a combination of 20% breadfruit starch and 80% wheat flour showed better proximate, functionality, and organoleptic attributes compared to 100% wheat flour noodles. This is a pivotal observation made that could give rise to better quality noodles infused with higher protein, fiber and substantial amounts of vitamins and minerals that food manufacturers can consider to formulate in the near future. However, the same study highlighted that 100% breadfruit flour noodles experienced lower sensory scores (undesirable slipperiness and glossiness) but better cooking yield (107). Thus, greater effort needs to be put into developing targeted post-processing technologies to improve the functionality of breadfruit starch for use in different carbohydrate staples. Porridges have been made using breadfruit flour as well with Mayaki et al. (108) optimizing the processing parameters to produce acceptable tasting variants.

### 5.3. Beverages and dairy products

Breadfruit flour is an optimal ingredient in the beverage industry. Probiotic beverages made using breadfruit flour (as the substrate) and various lactic acid bacteria as the starter bacteria has been explored by Gao et al. (60). It was observed that 7% breadfruit flour, 1% inoculum, and 15% sugar after fermentation at 30^°^C for 48 h produced the best tasting beverage. The beverage was characterized by panelists as having a fruity flavor, pale yellow color, and sour, and sweet taste (60). Breadfruit milk alternatives have also been tested with pasteurization for 30 min at 62^°^C producing the most favorable product with an acceptable shelf life (2). Probiotic yogurt with soy milk and 4% breadfruit flour with *Bifidobacterium bifidum* (ATCC 11883) and *Lactobacillus acidophilus* have been explored by Barber et al. (109). Though sensory attributes were not tested for, they optimized the fermentation parameters, minimum breadfruit flour amount and showcased cell count viability for a probiotic product. This is a promising area to work on and more research can be done to further optimize the processes, taste and quality of these beverages with the incorporation of breadfruit flour.

### 5.4. Meat analogs

Meat analogs are becoming more popular in the recent years due to the increasing awareness of animal welfare and environmental sustainability in curbing global warming (110). Meat analogs, also known as meat alternatives, are made from plant-based products to imitate the look, flavor and texture of meat (111). The functionality of breadfruit flour has been studied and have shown promising results. Extrusion cooking of breadfruit flour through extrusion cooking have shown promising results in its use in meat emulsion products such sausages and bologna (112). Soy protein gels incorporated with 4% extruded breadfruit flour did not affect processing yields and helped improve color and texture of protein/starch food systems (56). Similarly, beef meat emulsions combined with 3% extruded breadfruit flour did not affect the redness value of cooked meat emulsions or result in any cooking loss. Instead, a significant reduction in textural hardness was reported (57). Additionally, Huang et al. (57) found that increasing extruded breadfruit flour concentration significantly reduced hardness and chewiness of meat emulsions. Hafid et al. (113) showed that 50% tapioca flour substitution with breadfruit flour resulted in minimal cooking loss and greater chicken nugget yield without significantly affecting the protein and fat content of the nuggets. Since meat analogs are fairly new to the food industry, and the debate on whether these alternative protein sources are nutritive to human health are still underway, utilizing breadfruit flour as a functional ingredient in the production of meat analogs may be favorable.

## 6. Conclusion

With breadfruit gaining traction globally as a productive and highly nutritive crop, it is important to understand its food applications and health benefits to consumers. This review serves as a stimulus to further exploit and enhance the utilization of breadfruit worldwide. It has consolidated the current methods involved in the harvesting, post-processing of breadfruit and its use in various industrial food applications. The nutritional, functional, technological and physicochemical properties of breadfruit have shown to positively influence the production of healthier food products using breadfruit flour and starch. Breadfruit flour has also been known as a healthier alternative to other starches. It is a highly versatile commodity and has the potential to promote climate resilience and sustainability especially in low latitude agriculture systems. Therefore, there is a need for more awareness of this underutilized superfood to be made to the food industries to innovate and incorporate breadfruit into food applications. Future research can focus on the functionality of post-processed breadfruit and its flour, and for more targeted use in food applications.

## Author contributions

KM: conceptualization, writing—original draft, and writing—review and editing. YQ: writing—review and editing. CH: conceptualization, writing—review and editing, and funding acquisition. All authors contributed to the article and approved the submitted version.
